# Real‐World clinical features and survival outcomes associated with primary gastrointestinal natural killer/T‐cell lymphoma from 1999 to 2020

**DOI:** 10.1002/cam4.5136

**Published:** 2022-09-17

**Authors:** Yang Chunli, Jiang Ming, Ma Ziyan, Ji Jie, Lv Shuli, Huang Jie, Wu Yu, Xu Caigang, Zou Liqun

**Affiliations:** ^1^ State Key Laboratory of Biotherapy and Cancer Center West China Hospital, Sichuan University Chengdu China; ^2^ Department of Oncology West China Hospital, Sichuan University Chengdu China; ^3^ Department of Global Public Health Karolinska Institute Stockholm Sweden; ^4^ Department of Hematology West China Hospital, Sichuan University Chengdu China; ^5^ Lymphoma Project Group West China Hospital, Sichuan University Chengdu China

**Keywords:** extranodal NK/T‐cell lymphoma, gastrointestinal, nomogram, prognosis, survival

## Abstract

**Background:**

Primary gastrointestinal natural killer (NK)/T‐cell lymphoma (PGINKTL) is a rare T‐/NK‐cell lymphoma subtype, and the clinical features and survival outcomes remain largely unknown.

**Methods:**

To summarize the clinical features and survival outcomes of PGINKTL, PGINKTL cases diagnosed at our hospital from May 1999 to December 2020 were reviewed; and the clinical data, information on treatment strategies, and survival were collected. Survival analysis was performed using the Kaplan–Meier method and multivariable Cox proportional hazards regression. We constructed a nomogram to visualize the survival prediction of PGINKTL. The discriminative ability and calibration of the nomogram for prediction were tested using the concordance index (C‐index) and calibration plots.

**Results:**

The cohort included 81 cases, the median age was 36 years (range, 7–80 years), and the male‐to‐female ratio was 1.7:1. The most common clinical symptom at the time of diagnosis was abdominal pain (71.6%). The most common lesion site was the colon (59.3%). During a median follow‐up period of 37.7 months, the median overall survival (OS) time of 81 patients was 4.0 months (95% confidence interval [CI], 3.1–4.9 months), and the 2‐year OS rate was 30.7% (95% CI, 20.3%–40.1%). The multivariate analyses indicated that patients with an Eastern Cooperative Oncology Group (ECOG) performance status (PS) score ≥2, serum lactic dehydrogenase (LDH) level ≥ the upper limit normal (ULN), and perforation had worse OS. We used these data to establish a nomogram to predict survival for PGINKTL. The nomogram displayed good accuracy, with a C‐index of 0.726.

**Conclusion:**

The clinical features and poor outcomes of PGINKTL, which is a rare and fatal lymphoma type, are presented. The proposed nomogram provides an individualized estimate of survival for these patients. In the future, the study focused on exploring a better treatment strategy to improve survival is required in PGINKTL.

## Graphical abstract

This article investigated the clinical characteristics and survival outcomes of a rare lymphoma subtype, primary gastrointestinal natural killer (NK)/T‐cell lymphoma. To date, this is the largest series of cases reported at a single medical center. The median OS was 4.0 months and a 2‐year OS rate of 30.7%. Moreover, we observed that perforation, serum LDH ≥ULN and EOCG PS score ≥2 were markedly associated with worse OS.

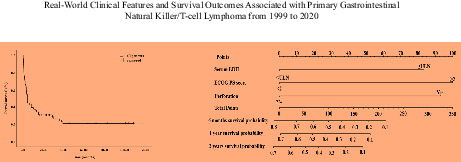

## 1 INTRODUCTION

Extranodal NK/T‐cell lymphoma (ENKTL) is an aggressive lymphoma that frequently occurs in the upper aerodigestive tract (UAT) in Asians, and the indigenous populations in Mexico, Central America, and South America.^1^, ^2^, ^3^ In contrast, primary gastrointestinal NK/T‐cell lymphoma (PGINKTL) has been insufficiently studied because of its low incidence and diagnostic difficulty. The incidence of PGINKTL was observed in 8% (84/1010) of patients with primary gastrointestinal (GI) tract lymphoma,^4^ 34.2% (13/38) of patients with primary gastrointestinal T/NK‐cell lymphoma (GI‐TNKL),^5^ and 19.6% (18/92) of patients with nonupper aerodigestive tract ENKTL.^6^

Radiotherapy combined with asparaginase‐containing chemotherapy is an established paradigm for early‐stage ENKTL treatment, resulting in 5‐year progression‐free survival (PFS) and overall survival (OS) rates of 64%–83% and 64%–89%, respectively.^7^, ^8^ For advanced‐stage ENKTL, asparaginase‐containing chemotherapy is frequently administered, resulting in a 3‐year PFS rate of 49.2% and a 3‐year OS rate of 33%–49.4%.^9^, ^10^ Currently, there is no standard treatment strategy for PGINKTL, clinicians often refer to treatment for ENKTL, nasal type, and surgery for some patients; however, their value for treating PGINKTL remains unknown. Conversely, the survival outcomes of gastrointestinal NK/T‐cell lymphoma (GINKTL) seemed significantly worse than those of UAT‐ENKTL. In 2013, Kim et al. summarized 81 ENKTL cases with intestinal involvement; the median overall survival time was 7.8 months.^11^ In 2021, a Korean group reported a median OS of 5.1 months for 13 patients with PGINKTL.^5^ Furthermore, an OS of 6 months was observed in a pooled database of 126 cases of PGINKTL.^12^ However, there are limited data available to summarize the clinical features, survival outcomes, and prognostic factors of this rare lymphoma.

During this study, we collected the data of 81 cases of PGINKTL diagnosed at our center from 1999 to 2020 to summarize the clinical manifestations, analyze survival outcomes, identify the factors that influence OS, and develop a nomogram to predict survival associated with PGINKTL.

## 2 MATERIALS AND METHODS

### 2.1 Patients

Between May 1999 and December 2020, we retrospectively reviewed 81 PGINKTL cases diagnosed at the West China Hospital of Sichuan University. The pathological diagnosis was confirmed by expert hematopathologists according to the World Health Organization (WHO) classification of hematopoietic and lymphoid tumors. PGINKTL is defined as lesions predominantly located in the gastrointestinal tract with or without regional lymph node involvement. Clinical data and survival information were obtained, including patient demographics, symptoms, lesion sites, Eastern Cooperative Oncology Group (ECOG) performance status (PS) score, serum lactic dehydrogenase (LDH) level, celiac lymph node involvement, plasma Epstein–Barr virus DNA copies, and therapy modality. Cases were staged using the Lugano staging system for primary gastrointestinal lymphoma. Contrast‐enhanced computed tomography or ^18^F‐2‐fluoro‐2‐deoxy‐D‐glucose positron emission tomography/computed tomography (^18^F‐FDG PET/CT) was administered to assist staging. All patients performed bone marrow aspiration and biopsy. Lesions appeared at multiple sites in more than one anatomical location (stomach, duodenum, jejunum/ileum, ileocecal, colon, and rectum). Multifocal site lesions appeared at more than one site in one anatomic location. OS was calculated using the date of diagnosis and the date of death for any reason or the last follow‐up date. The study was done under regulatory requirements and approved by the Ethics Committee of the West China Hospital of Sichuan University.

### 2.2 Statistical analysis

Univariate and multivariate analyses related to survival were performed using the Kaplan–Meier method, Cox proportional hazard model, and log‐rank test. In the multivariate analysis, factors with *p* < 0.05 from the univariate analysis results were included; and the forward stepwise selection was adopted. Add a variable with *p* < 0.05 and remove it with *p* > 0.1 for considering the collinearity between the variables used in this Cox model. Assumptions underlying the use of the Cox model were checked by observing curve crossover for every variable included in the Cox model, and no curve crossover means the assumption acceptance. The discriminative ability and calibration of the nomogram for prediction were tested using the C‐index and calibration plots. A calibration plot based on 1000 times bootstrapping resampling was generated to assess consistency among the observed and predicted 6‐month, 1‐year, and 2‐year survival outcomes. The *x*‐axis of the calibration plot represents the frequency distribution of predicted survival. The *y*‐axis represents the observed survival rate. The C‐index is a metric used to evaluate the model discrimination ability. In general, a C‐index of 0.5 means the model failed to discriminate the outcome of interest, whereas a C‐index of 1 indicates a perfect prediction model. A C‐index of approximately 0.7 (the “rule of thumb”) is an acceptable metric for a prediction model, particularly for rare diseases. All data were analyzed using SPSS (version 25.0; IBM Corp., Armonk, NY) and R software (version 4.1.1). The significance level was set at α = 0.05. Two‐tailed hypotheses tests were also conducted.

## 3 RESULTS

### 3.1 Patient characteristics

The characteristics of 81 patients are shown in Table 1. The median age was 36 years (range, 7–80 years), and 7.4% of the patients were older than 60 years. Of the 81 patients, 63% were men, and the male‐to‐female ratio was 1.7:1. A total of 30.9% of patients presented with a poor ECOG PS score (ECOG PS score ≥ 2). The most common clinical symptoms at diagnosis were abdominal pain (71.6%), anemia (69.1%), fever (66.7%), and hematochezia (53.1%). Additionally, the median time from the appearance of symptoms to diagnosis was 3 months (range, 9 days–60 months); 70.4% of cases were diagnosed within 6 months, and 11.1% were diagnosed after more than 12 months. The most common lesion site was the colon (59.3%), followed by the jejunum/ileum (30.9%), and ileocecal junction (30.9%). Additionally, 45.7% of the cases had multisite lesions and 54.3% with multifocal lesions. Celiac lymph node involvement was considered for 40.7% of cases. According to the Lugano staging system for primary gastrointestinal lymphoma,^13^ 48.2% of the cases were classified as stage I, 43.2% were classified as stage II, and 8.6% were classified as stage IV. Serum LDH data were obtained from 75 patients, and 51.9% had increased levels. Plasma Epstein–Barr virus DNA copies were detected in 23 patients; 65.2% of these copies were positive and 34.8% were negative.

**TABLE 1:** Clinical characteristics of patients with PGINKTL (*n* = 81)

Characteristics	*N* (%)
Age, years (median = 36, range = 7–80)	
>60 years	6 (7.4)
≤60 years	75 (92.6)
Sex	
Male	51 (63.0)
Female	30 (37.0)
ECOG PS score	
0–1	56 (69.1)
≥2	25 (30.9)
Clinical symptoms	
Abdominal pain	58 (71.6)
Hematochezia	43 (53.1)
Obstruction	2 (3.5)
Perforation	36 (44.4)
Diarrheal	28 (34.6)
Anemia	56 (69.1)
Fever	54 (66.7)
Time to diagnosis, months (median = 3, range = 0.03–60.00)	
Time from appearance to diagnosis	
<6 months	57 (70.4)
6 months to 1 year	15 (18.5)
>1 year	9 (11.1)
Pathological manifestations	
Ulcer	79 (97.5)
Mass	2 (2.5)
Location	
Stomach	1 (1.2)
Duodenum	3 (3.7)
Jejunum/Ileum	25 (30.9)
Ileocecal junction	25 (30.9)
Colon	48 (59.3)
Rectum	7 (8.6)
Multiple sites	37 (45.7)
Multifocal sites	44 (54.3)
Celiac lymph node involved	
Yes	33 (40.7)
No	48 (59.3)
Lugano stage	
I	39 (48.2)
II	35 (43.2)
IV	7 (8.6)
Serum LDH	
Increased	42 (51.9)
Normal	33 (40.7)
Unknown	6 (7.4)
Plasma EBV DNA copies	
Positive	15 (18.5)
Negative	8 (9.9)
Unknown	58 (71.6)

Abbreviations: EBV, Epstein–Barr Virus; ECOG PS score, Eastern Cooperative Oncology Group (ECOG) performance status (PS) score; PGINKTL, primary gastrointestinal natural killer (NK)/T‐cell lymphoma; serum LDH, serum lactic dehydrogenase level.

### 3.2 Morphologic and immunophenotypic features

Of the 81 patients, 97.5% had ulcerative lesions; two patients had a tumor mass convex to the intestinal lumen. Local necrosis was frequently observed. Most of the tumor cells in these specimens were medium to large, and several had small or medium, angiocentric, or angiodestructive features. Immunohistochemically (Table 2), tumor cells from all cases were positive for CD3ε and EBER, and 98.8% of cases were positive for the cytotoxic marker T‐cell intracellular antigen‐1/granzyme B. Positive CD56 immunostaining was observed in 72.8% (59/81) of cases. CD30 was detected with immunostaining in 30 patients; of these, 80% of the test results were positive; however, we did not calculate the exact expression value of CD30. The T‐cell receptor γ gene rearrangement was detected in 29 patients, and 37.9% (11/29) of patients demonstrated monoclonal rearrangement with the T‐cell receptor γδ phenotype. The median Ki‐67 proliferation index was 60% (range, 30%–95%) in this cohort.

**TABLE 2:** Immunophenotypic features of PGINKTL patients (*n* = 81)

Markers	No. of positive	%
CD3ε	81	81/81,100
CD4	2	2/17, 11.8
CD8	3	3/18, 16.7
CD20	2	2/80, 2.5
CD30	24	24/30, 80
CD56	59	59/81, 72.8
TIA/Granzyme B	80	80/81, 98.8
EBER	81	81/81, 100
TCR γ gene rearrangement	11	11/29, 37.9
Ki‐67, % (median = 60, range = 30–95)		

Abbreviations: CD, cluster of differentiation; EBER, Epstein–Barr virus‐encoded small RNAs; PGINKTL, primary gastrointestinal natural killer (NK)/T‐cell lymphoma; TCR, T‐cell receptor; TIA, T‐cell intracellular antigen‐1.

### 3.3 Treatment and survival outcomes

The initial therapy modality used for the 81 cases was reviewed; 67.9% were treated with surgery, 51.9% were treated with chemotherapy, and 6.2% were treated with radiotherapy as the initial therapy (Table 3). In contrast, 23.5% of the patients underwent surgery and chemotherapy, one patient underwent chemotherapy and radiotherapy, three patients underwent autologous hematopoietic stem cell transplantation, and nine patients received no therapy. Of the patients who underwent surgery, 23 underwent surgery before intestinal perforation, and 32 underwent surgery after perforation.

**TABLE 3:** Initial treatment modality of patients with PGINKTL (*n* = 81)

Therapy options	*N* (%)
Surgery	55 (67.9)
Chemotherapy	42 (51.9)
Chemotherapy with asparaginase	32 (39.5)
Chemotherapy without asparaginase	10 (12.3)
Radiotherapy	5 (6.2)
Surgery + Chemotherapy	19 (23.5)
Surgery + Chemotherapy + Radiotherapy	1 (1.2)
Chemotherapy + Radiotherapy	4 (4.9)
ASCT	3 (3.7)
Untreated	9 (11.1)

*Note*: Asparaginase drugs include pegaspargase or L‐asparaginase.

Abbreviations: ASCT, auto hematopoietic stem cell transplantation; PGINKTL, primary gastrointestinal natural killer (NK)/T‐cell lymphoma.

**FIGURE 1:**
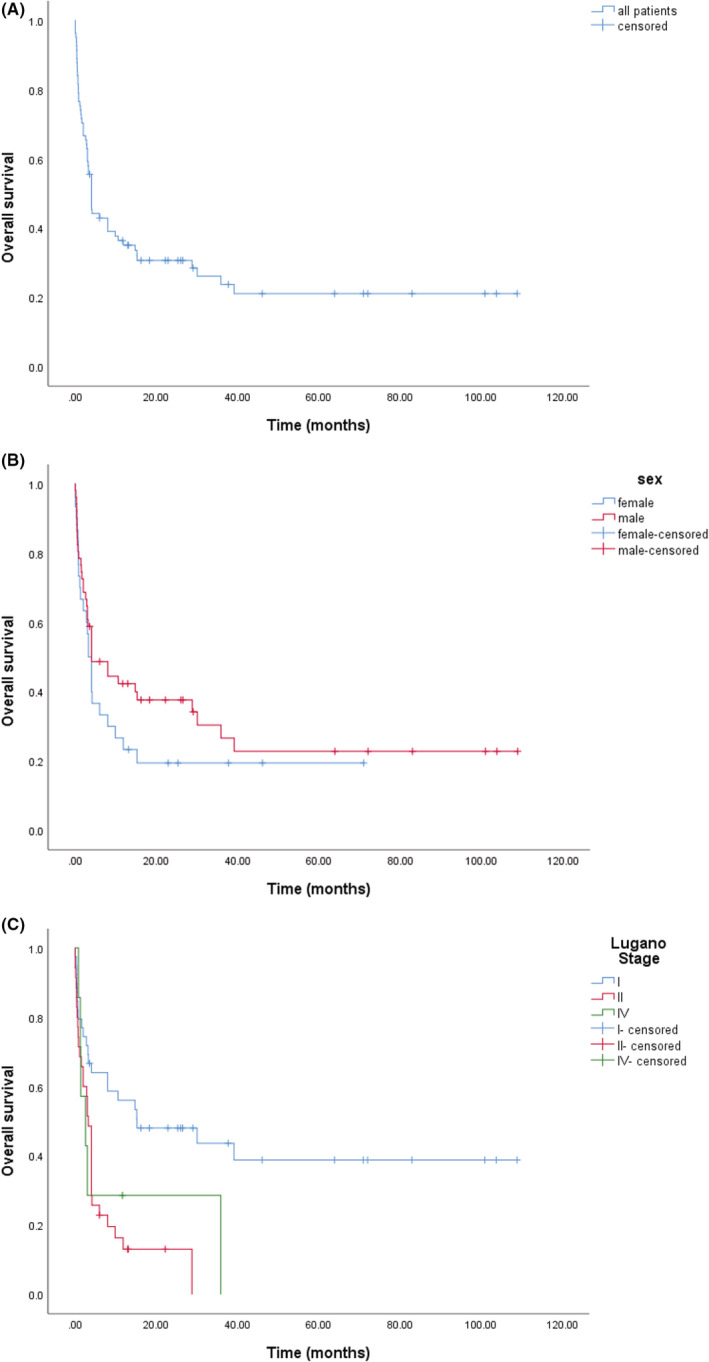
Kaplan–Meier survival curve of overall survival (OS) for primary gastrointestinal natural killer (NK)/T‐cell lymphoma patients. (A) The median OS was 4.0 months (95% confidence interval [CI], 3.1–4.9 months) for a total of 81 patients. The 2‐year OS rate was 30.7% (95% CI, 20.3–40.1%). (B) The median OS was 4.0 months (*n* = 51, 95% CI, 0.0–8.7 months) for men and 3.2 months for women (*n* = 30, 95% CI, 2.1–4.3 months), *p* = 0.242. (C) The median OS for patients with Lugano stage I was 15.2 months (*n* = 39, 95% CI, 0.0–40.2 months), 3.2 months (*n* = 35, 95% CI, 2.5–3.9 months) for stage II, and 2.6 months (*n* = 7, 95% CI, 0.0–5.6 months) for stage IV, *p* = 0.001.

The cutoff date for this analysis was October 20, 2021; 59 deaths occurred. After a median follow‐up period of 37.7 months (95% CI, 12.3–63.1 months), the median OS of the 81 patients was 4.0 months (95% CI, 3.1–4.9 months) and the 2‐year OS rate was 30.7% (95% CI, 20.3%–40.1%) (Figure 1A). We observed no significant difference in the OS of men (4.0 months; 95% CI, 0.0–8.7 months) and women (3.2 months; 95% CI, 2.1–4.3 months; *p* = 0.242) (Figure 1B). In this cohort, the median OS of patients with stage I was 15.2 months (95% CI, 0.0–40.2 months); however, it was 3.2 months (95% CI, 2.5–3.9 months) for those with stage II, and 2.6 months (95% CI, 0.0–5.6 months) for those with stage IV according to the Lugano staging system for primary gastrointestinal lymphoma (*p* = 0.001) (Figure 1C). Of note, 17.9% of patients (7/39) with stage I were still alive at 5 years or more after diagnosis (range, 63.9–108.9 months); however, no patients with stage II or stage IV were still alive at 5 years after diagnosis (data not shown).

Additionally, univariate and multivariate analyses were performed. The univariate analysis results (Table 4) indicated that an ECOG PS score ≥2 (*p* = 0.000), Lugano stage II or stage IV (*p* = 0.001), increased serum LDH (*p* = 0.000), celiac lymph node involvement (*p* = 0.015), perforation (*p* = 0.001), and anemia (*p* = 0.005) were significantly associated with worse OS. The multivariate analyses (Table 5) indicated that patients with an ECOG PS score ≥2 (relative risk [RR], 3.813; 95% CI, 2.094–6.943; *p* = 0.000), serum LDH ≥ the upper limit of normal (ULN) (RR, 2.238; 95% CI, 1.277–3.923; *p* = 0.004), and perforation (RR, 2.542; 95% CI, 1.466–4.409; *p* = 0.001) had worse OS. The maximum variance inflation factor (VIF) for every predictor in the Cox model was <1.11, which showed the nonexistence of multicollinearity.

**TABLE 4:** Univariate analysis of OS in patients with PGINKTL (*n* = 81)

Variables	Median OS, months	*p* value
Age		0.477
>60 years	2.563	
≤60 years	4.008	
Sex		0.242
Male	4.008	
Female	3.220	
ECOG PS score		**0.000**
≥2	0.723	
<2	8.016	
Lugano stage		**0.001**
I	15.211	
II	3.220	
IV	2.563	
Serum LDH^a^		**0.000**
≥ULN	2.004	
Normal	11.828	
Celiac LN involved		**0.015**
Yes	2.825	
No	8.016	
Fever		0.126
Yes	4.008	
No	10.579	
Perforation		**0.001**
Yes	2.004	
No	10.579	
Anemia		**0.005**
Yes	2.990	
No	14.752	
Multiple sites involved		0.754
Yes	4.008	
No	4.008	
Multifocal sites involved		0.171
Yes	4.008	
No	4.140	

The bold values presented in Table 4 means *p* < 0.05. Abbreviations: ECOG PS score, Eastern Cooperative Oncology Group (ECOG) performance status (PS) score; LN, lymph node; PGINKTL, primary gastrointestinal natural killer (NK)/T‐cell lymphoma; OS, overall survival; serum LDH, serum lactic dehydrogenase level; ULN, upper limit of normal.

^a^ 75 patients available.

**TABLE 5:** Multivariate analysis of OS in patients with PGINKTL (*n* = 81)

Variables	RR (95% CI)	*p* value
ECOG PS score ≥2	3.813 (2.094–6.943)	0.000
Serum LDH elevated	2.238 (1.277–3.923)	0.004
Perforation	2.542 (1.466–4.409)	0.001

Abbreviations: ECOG PS score, Eastern Cooperative Oncology Group (ECOG) performance status (PS) score; PGINKTL, primary gastrointestinal natural killer (NK)/T‐cell lymphoma; RR, relative risk; serum LDH, serum lactic dehydrogenase level.

### 3.4 The nomogram constructed and internal validation

**FIGURE 2:**
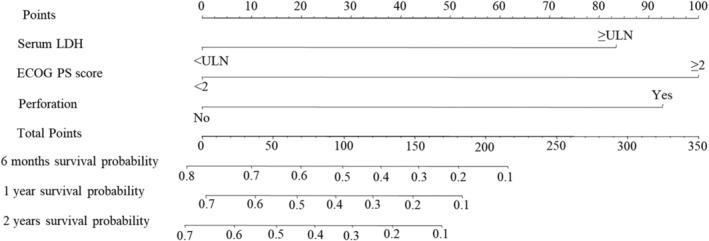
Nomogram to predict the survival probabilities in primary gastrointestinal natural killer (NK)/T‐cell lymphoma. The nomogram was constructed based on the following variables: serum LDH (lactic dehydrogenase), ECOG PS score (Eastern Cooperative Oncology Group performance status score), and perforation. The sum of these points is located on the total points axis, and a line is drawn downward to the survival axis to determine the probability of 6‐month, 1‐year, and 2‐year overall survival.

**FIGURE 3:**
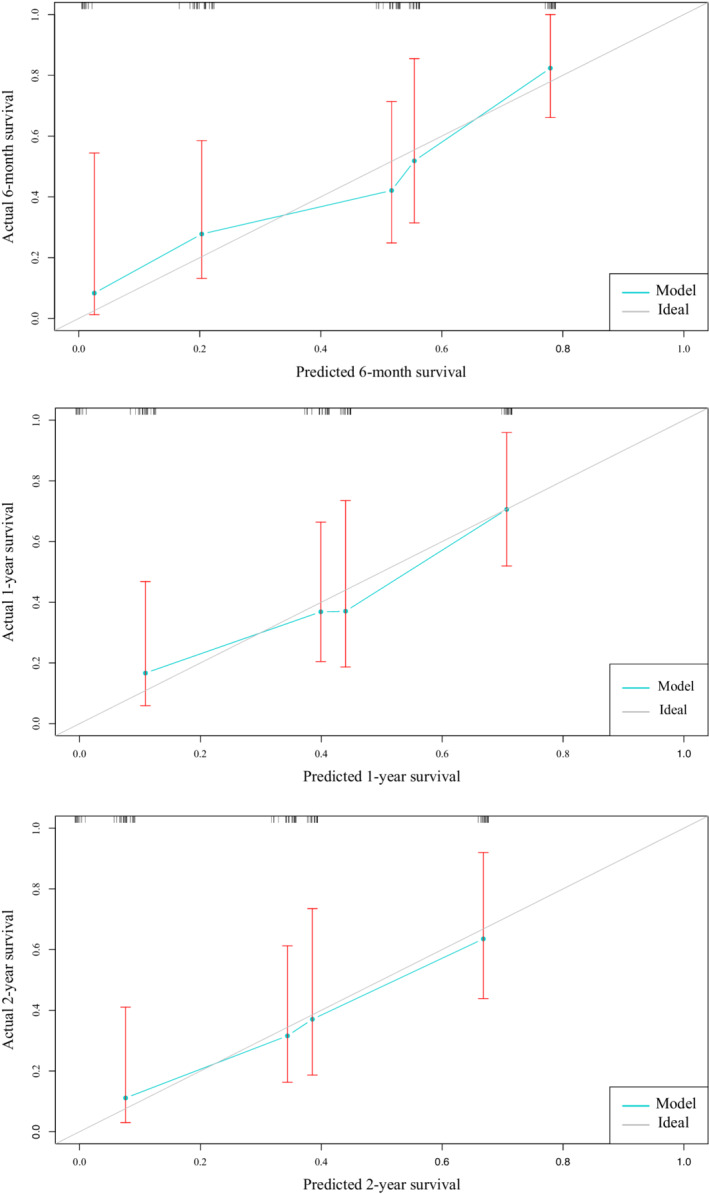
Calibration plots comparing predicted and actual survival in primary gastrointestinal natural killer (NK)/T‐cell lymphoma. The figure shows the actual against predicted 6‐month, 1‐year, and 2‐year survival probability of patients with PGINKTL, where the *x*‐axis shows the overall survival rate predicted by the nomogram, and the *y*‐axis represents the actual survival rate calculated by a Kaplan–Meier analysis.

The nomogram (Figure 2) included three variables (ECOG PS score, serum LDH, and perforation). It used regression model visualization to evaluate the survival probability associated with PGINKTL. After drawing a vertical line on every predictor, a corresponding point for each predictor appeared on the “points” axis. Summing all the points for each predictor on the “total points” axis allowed the user to predict the 6‐month, 1‐year, and 2‐year survival. For example, an individual with serum LDH < ULN, ECOG PS score ≥2, and perforation has a total score of 193 (0 + 100 + 93, respectively), resulting in a predicted 6‐month survival probability of approximately 17%; the predicted 1‐year and 2‐year survival rates were <10%. The C‐index for survival prediction for all patients was 0.726 according to internal validation. Additionally, the bias‐corrected curve on the calibration plot was close to the diagonal line (Figure 3), representing the consistency of the predicted observed survival with bootstrapping resampling.

## 4 DISCUSSION

ENKTL, nasal type is the most common T/NK‐cell lymphoma subtype in China^14^; however, PGINKTL is rare. To our knowledge, this is the largest series of cases reported at a single medical center. The data showed a poor prognosis for PGINKTL, with a median OS of 4.0 months and a 2‐year OS rate of 30.7%. Moreover, using the multivariate Cox proportional hazards model, we observed that perforation, serum LDH ≥ULN, and EOCG PS score ≥2 were markedly associated with worse OS; based on these data, a nomogram to predict the survival associated with PGINKTL was constructed.

The data showed that PGINKTL more frequently affected male patients 36–43 years of age. The most common involved site was the colon, followed by the jejunum/ileum and ileocecal junction, which is in agreement with the literature.^12^ In contrast, several studies reported that the small intestinal is most affected.^11^, ^15^ In this cohort, abdominal pain was the predominant symptom at the time of diagnosis, and an ulcerative lesion was the primary presentation during endoscopy and surgery, as described in the literature,^12^, ^15^ indicating that PGINKTL should be considered a differential diagnosis for ulcer diseases of the digestive tract, such as Crohn's disease. Additionally, based on the pathological results, 80% of PGINKTL patients had CD30 expression, suggesting that a target CD30 drug could be considered in some cases. Two studies reported that CD4 expression despite CD56 positive conferred a longer OS for PGINKTL^12^, ^15^; according to our data, only 2 of 17 patients positive for CD4 had an OS of 0.8 months and 1.5 months, respectively (data not shown).

Surgery is frequently performed when there is a high risk of perforation at the time of diagnosis or during the treatment and for symptom alleviation. In our study, 67.9% of patients were administered surgery, most were urgent surgery due to the acute intestinal perforation at the time of diagnosis. The prognosis role of surgery for intestinal lymphoma remains controversial.^16^, ^17^, ^18^ From this data, there was no OS benefit of surgery when stratified by surgery or not (Figure S1), as in our previous report.^19^ However, patients who underwent surgery before perforation had better OS than patients who underwent surgery after perforation (Figure S2), suggesting that the identification of cases with a high risk of perforation is critical. Nevertheless, the data necessary to identify these cases are lacking. Asparaginase (ASP) is the cornerstone of ENKTL treatment, we also assessed the role of ASP in PGNKTL. Thirty‐two patients with asparaginase‐based chemotherapy and 10 patients with nonasparaginase‐based chemotherapy were analyzed (Figure S3), and an improved OS trend for the asparaginase‐based chemotherapy group was observed. As the limited sample size, variation of chemotherapy regimens, and cycles in this cohort, further prospective studies with a large number of patients are still needed to confirm.

Increased serum LDH and high ECOG PS scores (≥2) are poor predictive survival markers of several lymphoma subtypes, including ENKTL, nasal type.^20^ Yang et al. developed a nomogram model to predict the probability of 5‐year OS for ENKTL, nasal type containing LDH and the ECOG PS score, it showed better performance than that of PINK model used for the validation cohort.^21^, ^22^ In this cohort, we found that these two markers were associated with worse OS in PGINKTL. Perforation is a serious life‐threatening complication of lymphomas involving the GI tract; several studies have reported worse outcomes for GI lymphomas.^23^, ^24^ Compared with B‐cell non‐Hodgkin lymphoma (hazard ratio, 6.31), T‐cell non‐Hodgkin lymphoma was associated with a higher risk of perforation (hazard ratio, 12.40; *p* < 0.0001).^25^ Furthermore, Vaidya et al. reported that 45% of GI‐involved lymphoma patients experienced perforation before chemotherapy.^25^ Our data showed that 94.4% of patients had perforation at the time of diagnosis. Patients with perforation had inferior OS in this cohort, 10.6 months vs 2.0 months, *p* = 0.001 (Supplementary Figure 4). We included these three factors in a nomogram to visualize the predicted survival of PGINKTL and validated it using calibration plots and C‐index; the predicted and practical outcomes were found to have acceptable coherence.

This study had several limitations. First, it was retrospective, and the study period was 21 years; therefore, selection and information biases could not have been avoided. Second, because PGINKTL is a rare tumor, the nomogram model needs to be validated by multicenter studies.

## 5 CONCLUSION

This study presented the clinical features and poor outcomes of a rare and fatal lymphoma type, PGINKTL. The proposed nomogram provides an individualized estimate of survival for patients with PGINKTL. In the future, the study focused on exploring a better treatment strategy to improve survival is required in PGINKTL.

## AUTHOR CONTRIBUTIONS

Zou L and Xu C: conception and design. Yang C, Jiang M, Ji J, Lv S, Huang J, and Wu Y: administrative support. Yang C and Ma Z: collection and analysis of data. All authors: manuscript writing, final approval of the manuscript, and accountability for work.

## FUNDING INFORMATION

This work was supported by the Sichuan Science and Technology Department for Key Research and Development projects (2019YFS0027).

## CONFLICT OF INTEREST

The authors declare that they have no conflict of interest to disclose.

## Supporting information


Figures S1‐S4

## Data Availability

The raw data supporting the conclusions of this article will be made available by the authors on reasonable request, without undue reservation." cd_value_code="text

## References

[1] Kwong YL . Natural killer‐cell malignancies: diagnosis and treatment. Leukemia. 2005;19:2186‐2194. 16179910 10.1038/sj.leu.2403955

[2] Avilés A , Díaz NR , Neri N , et al. Angiocentric nasal T/natural killer cell lymphoma: a single Centre study of prognostic factors in 108 patients. Clin Lab Haematol. 2000;22:215‐220. 11012633 10.1046/j.1365-2257.2000.00307.x

[3] Laurini JA , Perry AM , Boilesen E , et al. Classification of non‐Hodgkin lymphoma in central and South America: a review of 1028 cases. Blood. 2012;120:4795‐4801. 23086753 10.1182/blood-2012-07-440073

[4] Ding W , Zhao S , Wang J , et al. Gastrointestinal lymphoma in Southwest China: subtype distribution of 1,010 cases using the WHO (2008) classification in a single institution. Acta Haematol. 2016;135:21‐28. 26303279 10.1159/000437130

[5] Kim EK , Jang M , Yang WI , Yoon SO . Primary gastrointestinal T/NK cell lymphoma. Cancers. 2021;13:13. 10.3390/cancers13112679 PMC8199162 34072328

[6] Liu ZL , Bi XW , Zhang XW , et al. Characteristics, prognostic factors, and survival of patients with NK/T‐cell lymphoma of non‐upper aerodigestive tract: a 17‐year single‐center experience. Cancer Res Treat. 2019;51:1557‐1567. 30971067 10.4143/crt.2018.681 PMC6790852

[7] Zhang L , Jiang M , Xie L , et al. Five‐year analysis from phase 2 trial of "sandwich" chemoradiotherapy in newly diagnosed, stage IE to IIE, nasal type, extranodal natural killer/T‐cell lymphoma. Cancer Med. 2016;5:33‐40. 26633585 10.1002/cam4.569 PMC4708906

[8] Jeong SH . Extranodal NK/T cell lymphoma. Blood Res. 2020;55:S63‐S71. 32719179 10.5045/br.2020.S011 PMC7386895

[9] Fox CP , Civallero M , Ko Y‐H , et al. Survival outcomes of patients with extranodal natural‐killer T‐cell lymphoma: a prospective cohort study from the international T‐cell project. Lancet Haematol. 2020;7:e284‐e294. 32105608 10.1016/S2352-3026(19)30283-2

[10] Li J , Li J , Zhong M , Zhou H , Yu B . The clinical features and survival outcome of 107 newly diagnosed advanced stage Extranodal NK/T‐cell lymphoma cases: a triple‐center study. Cancer Manag Res. 2021;13:1541‐1549. 33623433 10.2147/CMAR.S292293 PMC7896804

[11] Kim SJ , Jung HA , Chuang S‐S , et al. Extranodal natural killer/T‐cell lymphoma involving the gastrointestinal tract: analysis of clinical features and outcomes from the Asia lymphoma study group. J Hematol Oncol. 2013;6:86. 24238138 10.1186/1756-8722-6-86 PMC4225665

[12] Haddad PA , Graham C . Survival determinants in primary intestinal NK/T‐cell lymphoma (PINKTCL): analysis of a pooled database. Blood. 2021;138:1396.

[13] Rohatiner A , d'Amore F , Coiffier B , et al. Report on a workshop convened to discuss the pathological and staging classifications of gastrointestinal tract lymphoma. Ann Oncol. 1994;5:397‐400. 8075046 10.1093/oxfordjournals.annonc.a058869

[14] Sun J , Yang Q , Lu Z , et al. Distribution of lymphoid neoplasms in China: analysis of 4,638 cases according to the World Health Organization classification. Am J Clin Pathol. 2012;138:429‐434. 22912361 10.1309/AJCP7YLTQPUSDQ5C

[15] Yu BH , Shui RH , Sheng WQ , et al. Primary intestinal Extranodal natural killer/T‐cell lymphoma, nasal type: a comprehensive clinicopathological analysis of 55 cases. PLoS One. 2016;11:e0161831. 27564014 10.1371/journal.pone.0161831 PMC5001693

[16] Cheung MC , Housri N , Ogilvie MP , Sola JE , Koniaris LG . Surgery does not adversely affect survival in primary gastrointestinal lymphoma. J Surg Oncol. 2009;100:59‐64. 19399785 10.1002/jso.21298

[17] Ahmed G , ElShafiey M , Abdelrahman H , et al. Surgery in perforated pediatric intestinal lymphoma. Eur J Surg Oncol. 2019;45:279‐283. 30224248 10.1016/j.ejso.2018.08.022

[18] Wang M , Ma S , Shi W , Zhang Y , Luo S , Hu Y . Surgery shows survival benefit in patients with primary intestinal diffuse large B‐cell lymphoma: a population‐based study. Cancer Med. 2021;10:3474‐3485. 33931950 10.1002/cam4.3882 PMC8124121

[19] Jiang M , Chen X , Yi Z , et al. Prognostic characteristics of gastrointestinal tract NK/T‐cell lymphoma: an analysis of 47 patients in China. J Clin Gastroenterol. 2013;47:e74‐e79. 23948755 10.1097/MCG.0b013e31829e444f

[20] Kim SJ , Yoon DH , Jaccard A , et al. A prognostic index for natural killer cell lymphoma after non‐anthracycline‐based treatment: a multicentre, retrospective analysis. Lancet Oncol. 2016;17:389‐400. 26873565 10.1016/S1470-2045(15)00533-1

[21] Yang Y , Zhang YJ , Zhu Y , et al. Prognostic nomogram for overall survival in previously untreated patients with extranodal NK/T‐cell lymphoma, nasal‐type: a multicenter study. Leukemia. 2015;29:1571‐1577. 25697894 10.1038/leu.2015.44

[22] Chen SY , Yang Y , Qi SN , et al. Validation of nomogram‐revised risk index and comparison with other models for extranodal nasal‐type NK/T‐cell lymphoma in the modern chemotherapy era: indication for prognostication and clinical decision‐making. Leukemia. 2021;35:130‐142. 32152465 10.1038/s41375-020-0791-3 PMC7787971

[23] Amer MH , El‐Akkad S. Gastrointestinal lymphoma in adults: clinical features and management of 300 cases. Gastroenterology. 1994;106:846‐858. 8143991 10.1016/0016-5085(94)90742-0

[24] Gou H‐F , Zang J , Jiang M , Yang Y , Cao D , Chen XC . Clinical prognostic analysis of 116 patients with primary intestinal non‐Hodgkin lymphoma. Med Oncol. 2012;29:227‐234. 21193968 10.1007/s12032-010-9783-x

[25] Vaidya R , Habermann TM , Donohue JH , et al. Bowel perforation in intestinal lymphoma: incidence and clinical features. Ann Oncol. 2013;24:2439‐2443. 23704194 10.1093/annonc/mdt188 PMC3755328

