# Supplementary material for: Real‐World clinical features and survival outcomes associated with primary gastrointestinal natural killer/T‐cell lymphoma from 1999 to 2020
Source: Cancer Med. 2022 Sep 17;12(3):2614–23. doi: 10.1002/cam4.5136 (PMC9939185; doi:10.1002/cam4.5136)

**Supplementary Figure 1. Kaplan-Meier survival curve of OS in patients with PGINKTL stratified by surgery (n=72).** The patients without surgery (n=17) had median OS of 15.2 months, and 4.0 months for those with surgery (n=55), *p*=0.151.


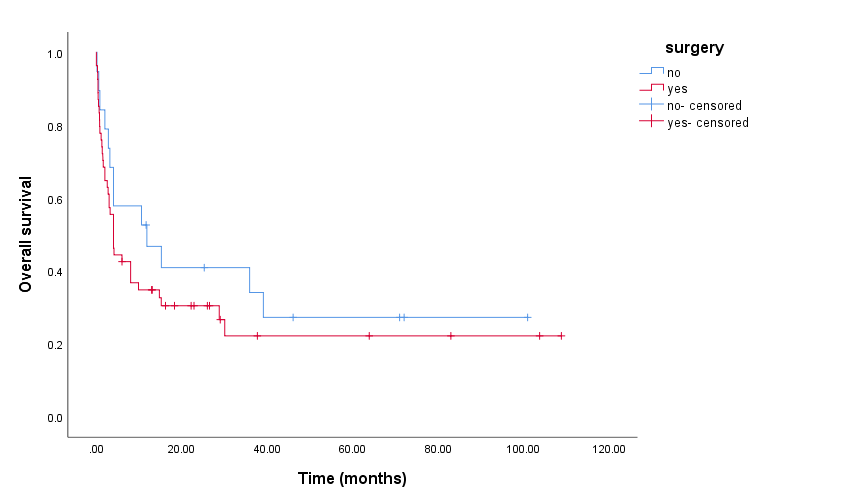


**Supplementary Figure 2. Kaplan-Meier survival curve of OS in patients with PGINKTL stratified by surgery timing (n=55).** A marked survival difference in patients with surgery before (n=23) and after perforation (n=32), the median OS was 9.9 months (95% CI not estimable), 2.8 months (95% CI, 1.0-4.5 months), *p*=0.009.


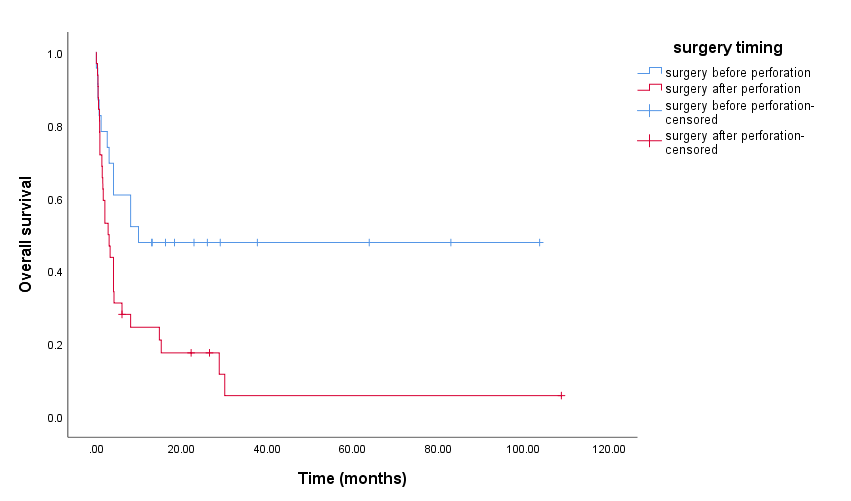


**Supplementary Figure 3. Kaplan-Meier survival curve of OS in patients with PGINKTL who received asparaginase-based chemotherapy (n=32).** There was no marked OS difference between the two groups, 35.9 months (asparaginase-based chemotherapy group) vs 4.0 months (non-asparaginase-based chemotherapy group), *p*=0.166.


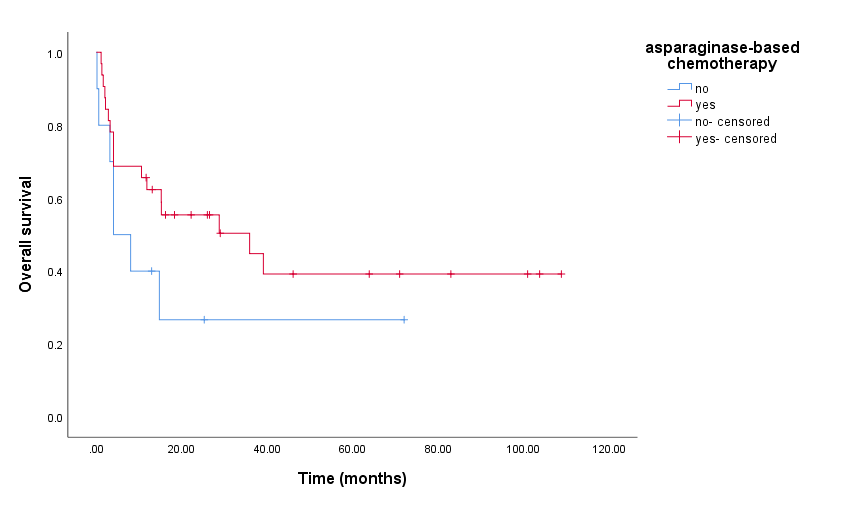


**Supplementary Figure 4. Kaplan-Meier survival curve of OS in patients with PGINKTL stratified by perforation (n=81).** Patients with perforation had worse OS in this cohort, 10.6 months (no perforation group) vs 2.0 months (perforation group), *p*=0.001


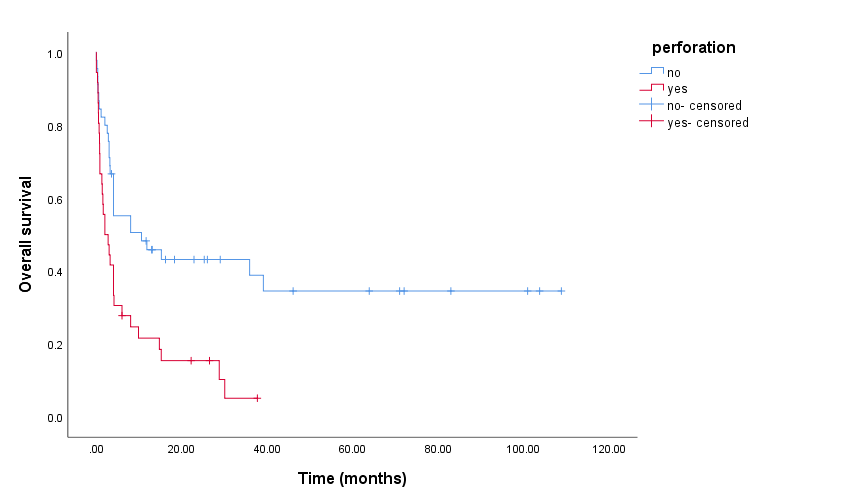

Supplement: Supplementary file 1 — Figures S1‐S4 [file CAM4-12-2614-s001.docx]
